# Self‐Assembled Inorganic Nanomembrane Tubes: Rolled‐Up Piezoelectrics for Microacoustic Wave‐Based Actuators and Sensors

**DOI:** 10.1002/adma.202512619

**Published:** 2025-11-28

**Authors:** Raphaël C. L‐M. Doineau, Christian N. Saggau, Stefan Baunack, Thomas Gemming, Yara Abdelaal, Robert Weser, Oliver G. Schmidt, Hagen Schmidt, Andreas Winkler, Mariana Medina‐Sánchez

**Affiliations:** ^1^ CIC nanoGUNE BRTA Tolosa Hiribidea 76 Donostia – San Sebastian E‐20018 Spain; ^2^ Chair of Micro‐ NanoSystems Center for Molecular Bioengineering (B CUBE) Dresden University of Technology Germany; ^3^ Leibniz Institute for Solid State and Materials Research Dresden Helmholtzstr. 20 01069 Dresden Germany; ^4^ GREMAN UMR 7347 Université de Tours CNRS INSA‐CVL Tours 37000 France; ^5^ DTU Electro Department of Electrical and Photonics Engineering Technical University of Denmark Orsteds Plads Building 340 Kongens Lyngby 2800 Denmark; ^6^ Center for Silicon Photonics for Optical Communications (SPOC) Technical University of Denmark Orsteds Plads Building 340 Kongens Lyngby 2800 Denmark; ^7^ Advanced NEMS Laboratory École Polytechnique Fedérale de Lausanne Route Cantonale Lausanne CH‐1015 Switzerland; ^8^ Research Center for Materials Architectures and Integration of Nanomembranes (MAIN) Chemnitz University of Technology 09126 Chemnitz Germany; ^9^ Ikerbasque Basque Foundation for Science Plaza Euskadi 5 Bilbao 48009 Spain

**Keywords:** 3D microstructure, dry release, microacoustic wave device, piezoelectric, rolled‐up, self‐assembly, thin‐film

## Abstract

Shaping piezoelectrics into innovative 3D microstructures is an emerging field, offering the potential to unlock new functionalities through topological engineering. Existing methods can create 3D piezoelectric composites and origami‐inspired structures, but they often reduce electromechanical resonance quality, especially when using organic elastic backbones with low mechanical quality factors. At the same time, 2D free‐standing piezoelectric nanomembranes used in acoustic wave resonators require thin film materials with low intrinsic stress to prevent device rupture as lateral dimensions increase. In this work, the first example of 3D self‐assembled piezoelectrics composed entirely of inorganic materials is presented. By precisely controlling mechanical stress and the nanomembrane release process, free‐standing nanomembranes are shaped into conformably stable tubular structures. The resulting rolled‐up piezoelectric (RUP) structures can be tuned in diameter, length, and winding number to optimize their performance for either actuation or sensing applications. Tubes up to 11 mm in length and 3.5 mm in rolling length are demonstrated, with functionality confirmed through 1‐port interdigital transducers (IDT) and 2‐port delay‐line architectures, integrating up to 10 mm^2^ of a free‐standing nanomembrane. Such devices can open new application possibilities for miniaturized medical devices, telecommunication, microfluidics, and energy harvesting, considering the large functional surface which adds another degree of freedom for topological design.

## Graphical abstract

This study demonstrates a self‐assembly process to generate free‐standing piezoelectric nanomembranes, forming ultracompact microtubular acoustic wave sensors and actuators. The miniaturized 3D piezoelectric platform reported in this work can be applied in telecommunication, energy harvesting, and acoustofluidics. Moreover, the 3D self‐assembly can add a degree of freedom for topological design of acousto‐optic and magneto‐acoustic devices.

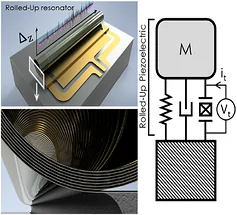

## 1 Introduction

Piezoelectric materials, which are highly sensitive and reversible electromechanical converters, have already demonstrated a strong technological impact in various fields as actuators for precision positioning or as generators/detectors for sensors such as gyroscopes or energy harvesters. The precision and efficiency of this electromechanical conversion phenomenon comes from the structural organization and properties of the material which is characterized by means of several variables: the piezoelectric charge constant “d” (C N^−1^) which defines the resulting charge to an applied strain, the electromechanical coupling coefficient “k” which corresponds to the fraction of electrical energy compared to mechanical energy of the acoustic wave, and the mechanical quality factor “Q_m_” which is related to the inherent loss or, in other words, characterizes the sharpness of the frequency characteristics of the device around its resonant frequency. Piezoelectrics can be either single crystalline, ceramic, or polymeric, which offer various advantages: Monocrystalline materials have a good compatibility with integrated circuits and semiconductor devices while reducing mechanical losses^[^ ^1^ ^]^ Ceramics offer advantages over single crystals including ease of fabrication into a variety of shapes and sizes, not constrained by crystallographic directions.^[^ ^2^, ^3^ ^]^ Piezoelectric polymers typically have a higher piezoelectric voltage constant (g_33_), which is key for sensors, and a low Young's modulus, compared to inorganic materials,.^[^ ^4^ ^]^ which enables processing flexibility for large scale manufacturing into a variety of size and shapes. However, while off‐resonance piezoelectric performance relies mostly on d_33_ and g_33_ piezoelectric coefficients, on‐resonance performance is driven by the electromechanical coupling coefficient (k_33_) and the mechanical quality factor (Q_m_).^[^ ^5^ ^]^ Consequently, inorganic materials with high mechanical quality factor and Young's modulus should be favored in the design of composite or multilayer devices when on‐resonance applications are considered.

Considering technological bottlenecks and manufacturing capabilities, those materials have mostly been used within planar interfaces, as emphasized in acousto‐optic modulation of photonic resonators^[^ ^6^ ^]^ and Lamb wave resonators^[^ ^7^ ^]^ research perspectives. Recently, efforts to build piezoelectric materials and composites, or more generally to architect flexible electronics in functional 3D structures, have emerged from the scientific community.^[^ ^8^, ^9^ ^]^ Structuring piezoelectric crystals into 3D architectures, can start with simple surface shaping to tailor the mechanical output of a piezoelectric transducer.^[^ ^10^ ^]^ More complex approaches have led to 3D printing approaches for energy harvesting,^[^ ^11^ ^]^ including optimized 3D composites with properties similar to the ones found in the raw material.^[^ ^2^ ^]^ Concomitant to the idea of flexible electronics and smart fabric, the fabrication of functional piezoelectric fibers has been reported in the literature,^[^ ^12^ ^]^ integrated into wearables, to demonstrate sensor applications such as ambient sound monitoring.

Besides, another approach of 3D structuring consisting in the release of a strained nanomembrane and its self‐assembly into a tubular structure was conceptualized more than twenty years ago,^[^ ^13^ ^]^ leading to many diverse applications, including among others microantennas,^[^ ^14^ ^]^ optical whispering gallery resonators,^[^ ^15^, ^16^, ^17^ ^]^ microcapacitors,^[^ ^16^, ^18^, ^19^ ^]^ microbatteries^[^ ^20^ ^]^ and Lab‐on‐a‐Chip sensing functions.^[^ ^21^ ^]^ Different sensing platforms, in particular the ones based on rolled‐up tubular structures, have been developed.^[^ ^22^, ^23^, ^24^ ^]^ This concept is based on the development of multifunctional rolled‐up electrodes through strain‐engineering of nanomembranes on top of a sacrificial layer. The sacrificial layer is then selectively etched, transforming a 2D architecture into a tube‐like device. In our group, we have developed high‐performance electrochemical sensors by integrating electrical transducers in such microtubes. The axial configuration enhances the sensing capabilities in microfluidic devices as the sensing surface per fluid volume and total capacitance increase, favoring in this way the signal coupling with the detection volume of the sample.^[^ ^25^ ^]^ For instance, our reported DNA biosensor^[^ ^26^ ^]^ showed superior sensitivity of four orders of magnitude compared to the equivalent planar counterparts, achieving attomolar detection levels of Avian Influenza Virus H1N1 DNA, without amplification or labeling. Moreover, the small radius of curvature from the tubular structures is favorable for microfluidic applications where low Reynolds numbers are required to obtain predictable laminar flow. Consequently, it would become particularly interesting for the miniaturization and automation of microfluidic devices to develop tubular piezoelectrics which can be used for fluid manipulation using the acoustofluidic phenomenon.

Finally, to further contextualize this study, it is important to mention that rolled‐up nanomembrane assembly was already applied to several crystalline materials for semiconductor applications, with a total thin film thickness usually below 100 nm, and demonstrated with materials such as InGaAs,^[^ ^27^ ^]^ VO_2_,^[^ ^28^ ^]^ Si.^[^ ^29^ ^]^ Rolled‐up nanomembranes made from aluminum nitride (AlN) were also previously demonstrated. However, those prior studies focused strictly on strain engineering studies or electrophoretic applications,^[^ ^30^, ^31^ ^]^ not considering their piezoelectric properties.a.

In this work, we focus on the electromechanical and more precisely the piezoelectric performance of 3D structured nanomembranes made of AlN, a III‐V semiconductor with a wide bandgap. We demonstrate here for the first time the feasibility of shaping large surfaces (up to 10 mm^2^) of a free‐standing piezoelectric nanomembrane into a tubular structure, obtaining the first “Rolled‐Up Piezoelectric” (RUP) to the best of our knowledge. Moreover, the fabrication process developed here enables the self‐assembly of textured polycrystalline inorganic piezoelectric thin films, with a thickness large enough to support plate wave excitation. The final devices exhibit adjustable dimensions and curvature, yielding a well‐defined footprint after self‐assembly. Their tubular geometry stabilizes the strained nanomembrane and provides a tunable inner confinement for subsequent acoustofluidic applications. Altogether, the here‐reported self‐assembled tubular piezoelectric structure, capable of supporting acoustic wave propagation has the potential for novel topology designs that may favor the fabrication of high‐performance chip‐scale energy harvesters and acoustofluidic devices. The platform additionally function as a research platform for exploring acousto‐optic^[^ ^6^ ^]^ and magneto‐acoustic^[^ ^32^, ^33^ ^]^ phenomena.

## 2 Results and Discussion

We describe here a functional method to release a piezoelectric thin film into a free‐standing nanomembrane, conformably stabilizing it in 3D as a tubular structure. The rolling of thin films into microtubes was conceptualized and experimentally demonstrated long ago^[^ ^13^, ^34^ ^]^ and is often referred to as “Rolled‐Up” nanomembrane devices. However, applying this tubular self‐assembly method to large‐area, with relatively thicker and highly crystalline materials, particularly for piezoelectric actuators and sensors, has not yet been demonstrated. Another important parameter was the choice of the piezoelectric material and the thin‐film fabrication process being compatible with the later described 3D self‐assembly. We opted here for aluminum nitride because AlN, as a III‐V wide bandgap semiconductor, exhibits good piezoelectricity properties under a hexagonal wurtzite crystal structure. Furthermore, AlN is a CMOS‐compatible material demonstrating higher stability in solution compared to zinc oxide (ZnO)^[^ ^35^ ^]^ which will turn to be particularly important for acoustofluidic applications.

**Figure 1:**
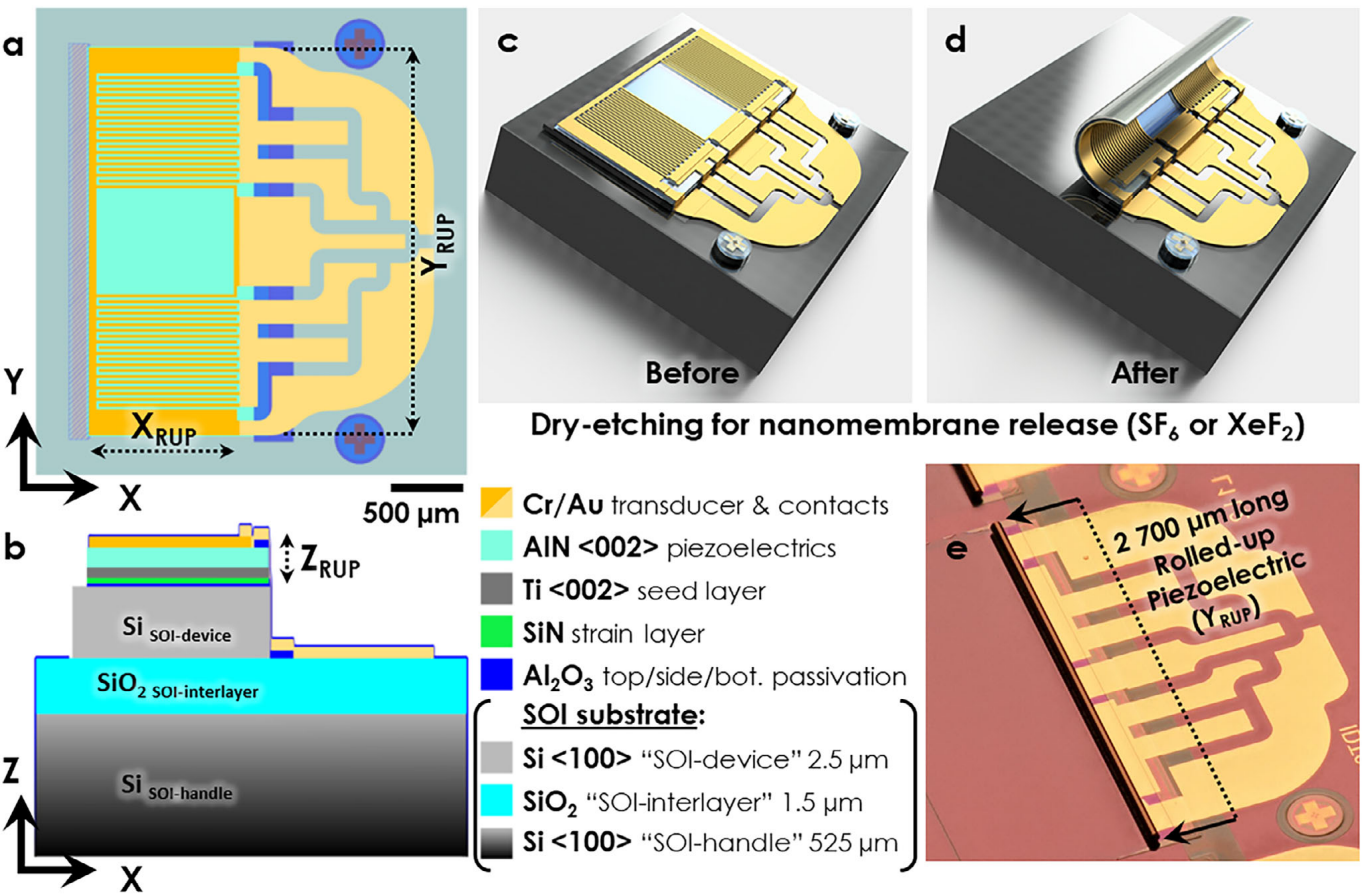
Rolled‐up piezoelectric platform fabrication: a,b) top view and cross‐section view of the thin‐film stack before membrane release and rolling. Layer thicknesses are not to scale along the *Z* axis for visual purposes. c,d) schematic illustration of the device before and after directional release of the thin‐film stack through dry‐etching of the Si <100> from the “SOI‐device” layer using either SF_6_ or XeF_2_ etchant. The rolling curvature is highly exaggerated for visual purposes. e) digital microscopy image of a final device (arrow pointing toward extremities of the rolled‐up structure, having here a 2700 µm tubular length).

The process developed to fabricate those RUP relies here on a multilayer assembly, patterning, and controlled release for the *de novo* free‐standing nanomembrane to curl under inherent strain into the desired tubular structure (**Figure** **1**). The approach has been inspired from a process previously developed in our laboratory to fabricate high‐quality microtubular optical resonators^[^ ^16^ ^]^ and is visually detailed in the supplementary information (Figure S1, Supporting Information). The method is based on a silicon sacrificial layer, which is dry etched to release  a nanomembrane, combined with a precise, ultra‐thin, and conformal passivation of the surfaces with atomic layer deposition of alumina.^[^ ^16^, ^18^ ^]^ This allows to overcome two major hurdles during the release of the strained nanomembranes, decreasing the risk of devicerupture during and after self‐assembly: first, thanks to dry‐etching, the free‐standing nanomembrane no longer suffers from liquid surface‐tension which may cause a complete collapse when drying device after self‐assembly; second, the minimally thick Al_2_O_3_ passivation layer allows simultaneously for efficient etchant protection (especially protecting SiN strain layer against SF_6_ or XeF_2_ attack during self‐assembly) and for its desired smooth ripping on both extremities of the tube (along the *X* axis) in order to gently release the nanomembrane from the substrat.

Here, the multilayer material stack was organized as follows: We opted for a silicon on insulator (SOI) substrate with the top Si <100>, usually called the “device layer”, acting here as a sacrificial layer and with the SiO_2_ buried thermal oxide, acting as an etch‐stop layer while being minimally attacked during isotropic SF_6_ or XeF_2_ etching. The material stack, which will be later released as free‐standing nanomembrane, consists of a SiN strained layer (with ≈−1.92 GPa compressive stress), a polycrystalline Ti seed layer, a textured piezoelectric AlN layer exhibiting tensile strain, a Cr/Au metallization for transducer electrodes and contact pads, and finally a full surface (bottom, sides and top) passivation layer of Al_2_O_3_. The directional release and self‐assembly is obtained by opening a window in the passivation layer, enabling the formation of a clean etch‐front and a uniaxial strain‐release, where the nanomembrane rolls under the formation of a tubular structure parallel to the etch‐front (Movie S1, Supporting Information). In the meantime, the ultra‐thin passivation layer (≈2 nm) was smoothly ripping on each extremities of the tubular structure while maintaining a linear etch‐front. The requirement for a perfect passivation is made visually clear at the end of the supplementary movie with the apparition of pinholes, quickly evolving into large defects. Passivation defects at the interface between the tube and electrical pads can be further avoided by a locally higher passivation thickness, which is additionally required to avoid an electrical short circuit between the electrodes and the Ti layer (Figure S1, Supporting Information: Step 7). Furthermore, the rolling curvature was systematically investigated and could be directly correlated to the thickness ratio of the tensile (Ti and AlN) over compressive (SiN) layers along the thickness of the multilayer nanomembrane (*Z_RUP_*) with an achieved minimal and maximal radius of curvature, respectively, of 32 and ≈ 750 µm (Figure S2, Supporting Information). A predictive control over the 3D RUP geometry is consequently possible, and the nanomembrane curvature could be estimated from the initial strains within the multilayer stack.^[^ ^36^ ^]^

Altogether, with this functional method, we have been able to manufacture devices with up to 10 mm^2^ of free‐standing piezoelectric nanomembrane, with the longest tubular structure (*Y_RUP_*) of 11 mm and a longest rolling length (*X_RUP_*) of 3.5 mm, with a functional thickness of piezoelectric thin film ranging between 0.1 to ≈1 µm, and with a yield of 8 out of 8 at chip scale (Figure 1 and Figures S2,S3, Supporting Information), offering a surface large enough to integrate multiple interdigital transducers (IDT) and sensor area. Remarkably, the mean length of functional free‐standing piezoelectrics is three to four orders of magnitude larger than the composite film thickness. Due to the nature of this first‐time study, the manufacturing yield is still limited, depending on the device design, given the limited sample size (SOI substrate cut into 13 mm × 11 mm samples). Consequently, in this study, with 8 out 8 structures per sample successfully rolling, we can claim that our fabrication yield is already above 87.5% (> 7/8). Furthermore, with a similar fabrication workflow further optimized here for larger and thicker nanomembranes, previously a yield of tubular nanomembrane successful self‐assembly > 98% on 10 × 10 mm substrate dies (n_structures_ = 299) or > 94% on 4″ wafer (n_structures _> 6000) as well as > 91% functional capacitor device was reported.^[^ ^16^ ^]^ For the present study, digital microscopy images from most of the chips fabricated and used to acquire the data of the present work are included in the supplementary material (Figures S2,S3, Supporting Information). They exemplify the successful self‐assembly of microstructures at a chip scale.

**Figure 2:**
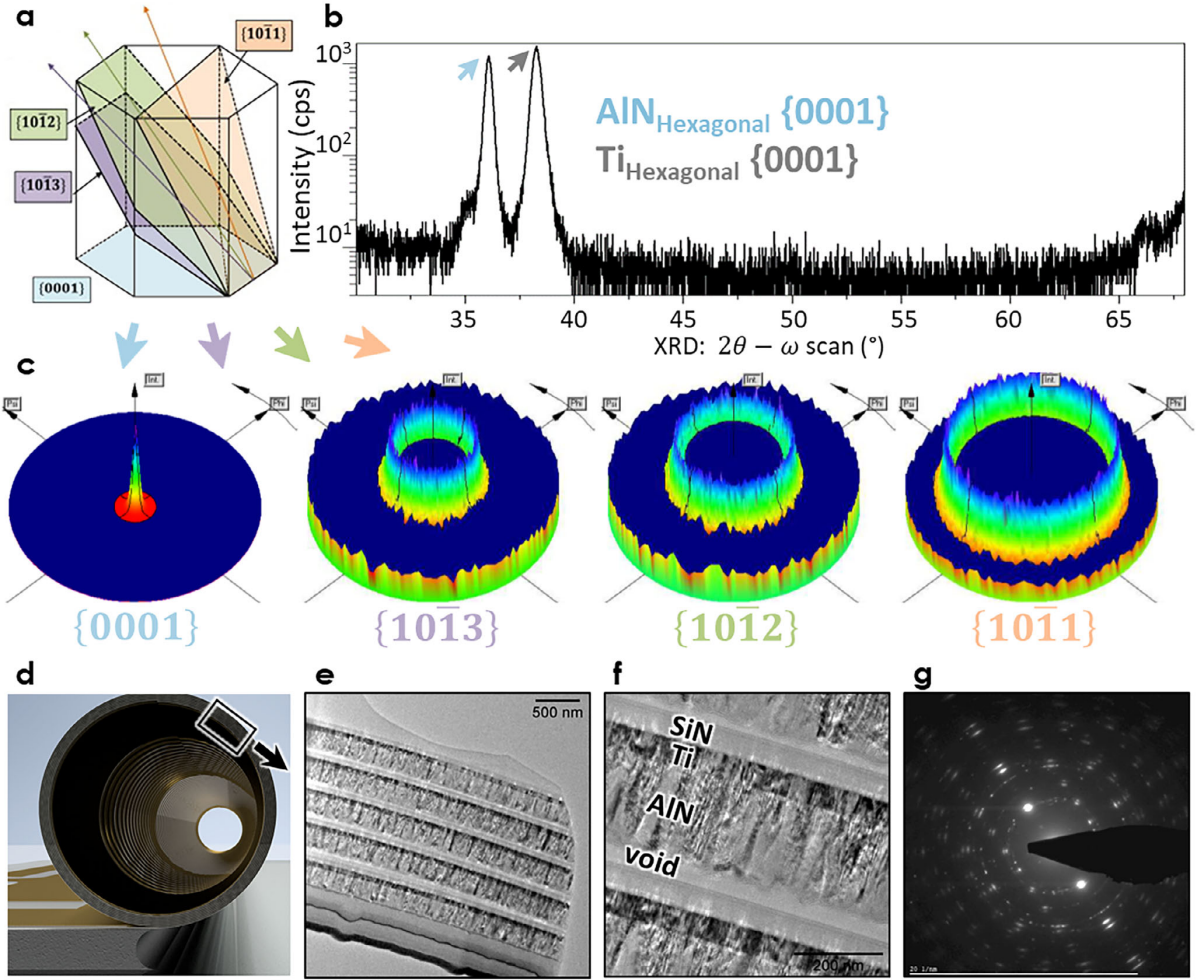
Characterization of the thin film stack: a) schematic illustration of the crystal planes orientation, b) XRD 2theta‐omega scan of the consecutively sputtered layers of Ti‐AlN on Si <100> substrate, c) XRD pole figures made at the 2‐theta angle related to crystal planes {0001}, $\left\{10\bar{1}3\right\}$, $\left\{10\bar{1}2\right\}$ and $\left\{10\bar{1}1\right\}$ respectively from left to right, d) schematic illustration of the multilayer rolled‐up and e,f) FIB‐cut lamella imaged with TEM bright field, (e) overview of 5 windings, (f) zoom‐in and layer identification within one winding, and (d) SAED pattern in the AlN layer shows the crystalline structure. Scales are respectively (c) linear intensity, (e) 500 nm, (f) 200 nm, and g) 20 1/nm.

Furthermore, we required a low‐temperature piezoelectric AlN deposition process to avoid affecting the strain built and preserved in the SiN layer, which is further required for 3D self‐assembly. We opted for a sputtering process, which will subsequently enable us to develop an industrially scalable manufacturing process with a low thermal budget, suitable for CMOS backend integration. Consequently, we developed a DC‐reactive sputtering process of aluminum under mixed argon and nitrogen gas flow. Sputtering parameters were optimized according to previous studies.^[^ ^37^, ^38^, ^39^ ^]^ To our view, the most critical parameters appeared to be the substrate and seed layer, especially with the surface roughness and crystalline texture. Therefore, we selected SOI substrates that offer a very low surface roughness compared to lab‐made sacrificial layers compatible with our method (i.e., polycrystalline Si layer (PECVD) or Mo layer (DC‐Sputtering)). The so‐called “device layer” of the SOI substrate was chosen with high conductivity (R < 5 Ω). Considering that the electric field plays an important role during the deposition, a conductive substrate or sample with metallization on top should be favorable for the crystallization process. Furthermore, we used a Ti seed layer sputtered in situ prior to AlN because of its favorable hexagonal texture.^[^ ^37^, ^38^ ^]^ The quality of our sputtering process to fabricate piezoelectric AlN layer on pure Si < 100 > substrates was first analyzed using X‐ray diffraction (XRD) (**Figure** **2**a–c) and compared to diffraction value from the ICDD database^[^ ^40^ ^]^ (AlN and Ti hexagonal PDF cards being respectively 00‐066‐0534 and 00‐044‐1294). First, the 2Theta‐Omega scan confirmed the hexagonal and *c*‐axis‐preferred growth from both Ti and AlN layers (Figure 2b). To further characterize the quality of our AlN layer, we generated pole figures from the {0001}, $\left\{10\bar{1}3\right\}$, $\left\{10\bar{1}2\right\}$ and $\left\{10\bar{1}1\right\}$ AlN crystal orientations at respectively 2‐Theta 36.05°, 66.07°, 49.83° and 37.94°. The results showed a characteristic ring pattern from the $\left\{10\bar{1}3\right\}$, $\left\{10\bar{1}2\right\}$ and $\left\{10\bar{1}1\right\}$ planes which is typical for a fiber‐texture polycrystalline material (Figure 2c and Figure S4. Supporting Information). Furthermore, the pole figure for the {0001} plane showed a well‐oriented and narrow peak. Therefore, considering both results from the 2Theta‐Omega scan and pole figures, we can conclude that we obtained the desired wurtzite AlN thin film with a fiber texture, which meant that all grains were *c*‐axis‐orsiented out of plane but with random orientation of the hexagonal columns in‐plane. This *c*‐axis‐oriented fiber texture is particularly important for our later application, considering the dominant piezoelectric response of AlN is along the *c*‐axis. Additionally, the material quality after 3D self‐assembly was characterized by transmission electron microscopy analysis (TEM) of a lamella prepared by FIB‐cut (Figure 2d–g). A digital microscopy image of the rolled‐up device before FIB‐cut is available in the supporting information (Figure S2c, Supporting Information). TEM bright field images show five windings of the thin‐film stacks organized in the 3D tubular structure with AlN grains oriented radially to the tube. Thin‐film stack composition after self‐assembly was monitored by STEM‐EDS (Figure S5, Supporting Information), and selected area electron diffraction analysis (SAED) further unveiled the crystallinity of AlN at the nanoscale (Figure 2g and Figure S6, Supporting Information).

**Figure 3:**
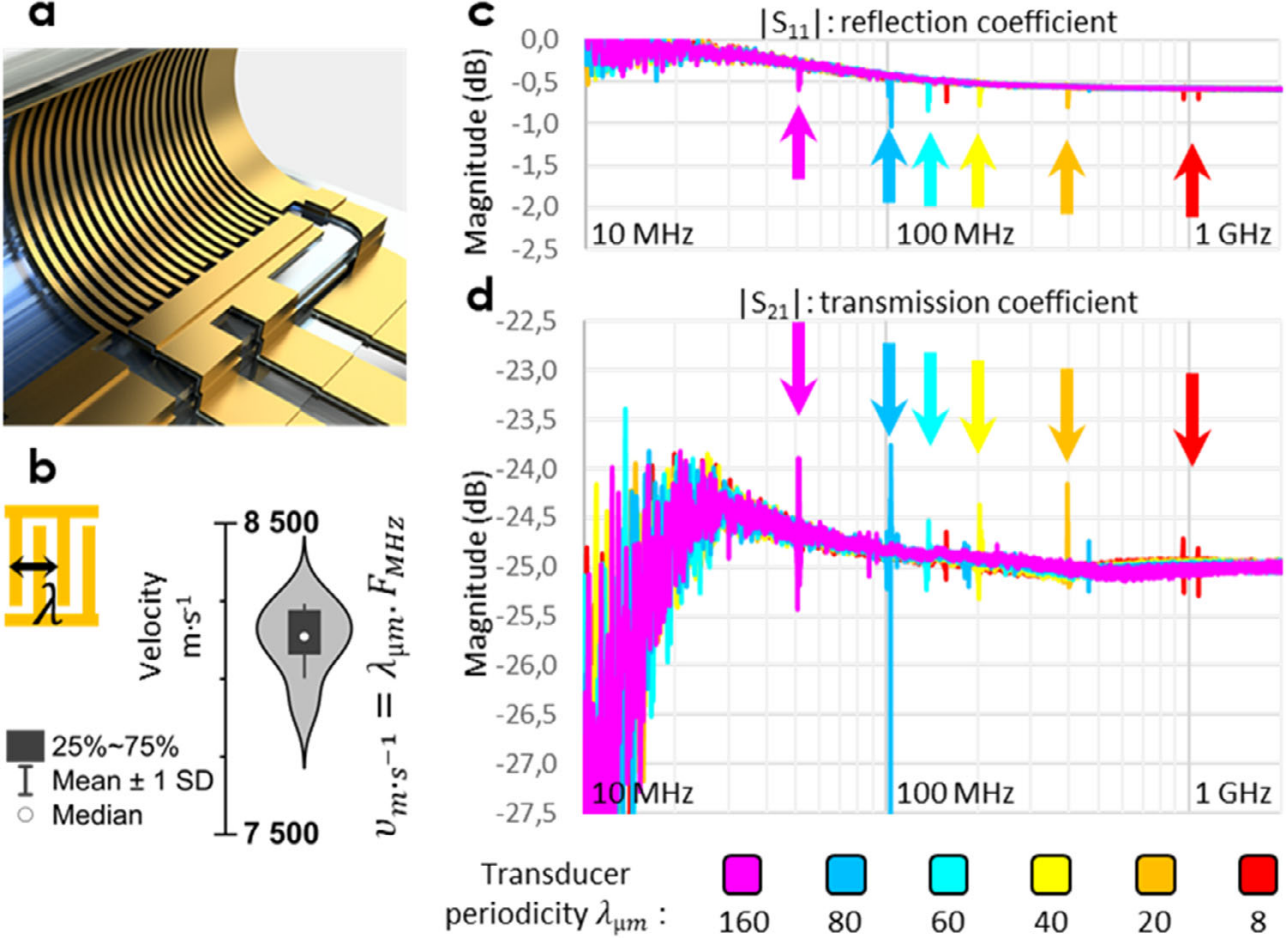
Analysis of the wavelength‐dependent responses of the devices: a) Illustration of the IDT integration into the rolled‐up device. b) Violin plot from the measured propagation velocities. Electrical characterization of the transducer's frequency characteristics in terms of c) reflection (S_11_) and d) transmission between IDTs (S_21_), depending on the transducer wavelength λ.

Following the successful fabrication of a textured AlN thin‐film and its 3D self‐assembly, we designed several IDTs to characterize their piezoelectric performance. Our primary IDT design (Figure 1a) consisted of two identical IDTs while applying a mirror symmetry at the middle of the tubular structure. With this 2‐port design, we could analyze RF scattering (S‐) parameters with a vector network analyzer and characterize the complex‐valued frequency‐dependent electrical reflection coefficient S_11_ and electric transmission coefficient between IDTs S_21_. The operating transduction frequency was identified with the scattering parameter magnitude |S_11_| and confirmed with the transmission between IDTs |S_21_| (**Figure** **3**). We used IDTs with a finger electrode periodicity λ of 80 µm (20 µm wide electrodes and gaps) exhibiting a resonance close to 100 MHz depending on the thickness ratio between the AlN and SiN layers. This variation from the operating frequency of the transducer was most likely batch dependent due to a minor change in both the AlN texture quality (Figure S6, Supporting Information) and the SiN residual strain affecting the overall mechanical tensor characteristics from the composite. To confirm the transduction behavior from our device, we designed a set of transducers by varying their IDT period $\lambda \in \left\{8,\;20,\;40,\;60,\;80,\;160\;\mu m\right\}$ (Figure 3b–d). Results highlighted a clear and linear correlation between IDT's wavelength and transduction frequency (Figure S7, Supporting Information), which was matching with the relation linking transduction frequency to wavelength (Equation 1) with ν the estimated sound velocity in the material.

(1) $$F_{res}=\frac{\nu }{\lambda }$$

The increased standard deviation observed on the sound velocity (Figure 3b) can be explained due to precision issue during film deposition, e.g., regarding film thickness or mechanic/electric property homogeneity, and IDT fabrication. Additionally, we estimated the mechanical quality factor (Q_m_) of the structures based on the vector network analyzer measurements. Considering the magnitude from the signal of interest, we opted for the following calculation of Q_m_ (Equation 2), with *f_res_* the response frequency of the structure and Δ*f* the full width at half maximum of response peak:

(2) $$Q_{m}=\frac{f_{res}}{\Delta f}$$

The analysis of the six different transducer structures (Figure 3c) allowed us to estimate a Q_m_ of 298 with a standard deviation of 45, which is comparable to Q_m_ values from ceramic piezoelectric materials,^[^ ^41^ ^]^ while it remains a relatively low quality factor compared to structures based on conventional inorganic piezoelectric materials, including reported AlN resonators.^[^ ^42^, ^43^ ^]^ However, on the one hand, it is higher than the one of piezoelectric polymers^[^ ^44^ ^]^ and on the other hand, the complex mechanical nature of the rolled‐up structure can further on be improved with respect to, e.g., layer‐to‐layer coupling or piezoelectric thin film properties. To complete this first set of electrical characterization, we performed two additional control experiments: first, we measured the same scattering parameters on a batch of devices with a constrained piezoelectric nanomembrane (i.e.,: multilayer stack before release and 3D self‐assembly), and secondly, we measured the scattering parameters of a rolled‐up device without piezoelectric properties while producing a non‐textured amorphous AlN layer (see respectively Figures S8–S10, Supporting Information). The transduction behavior completely disappeared from the control experiments, highlighting the requirement for a free‐standing and piezoelectric tubular structure to obtain a functional device. The lack of measurable acoustic excitation behavior of devices with a constrained nanomembrane, meaning that the unreleased multilayer composite remained solidly mounted (i.e., clamped) to the substrate, is expected to arise from the strong difference in the effective thickness of the device. On one side, our self‐assembled devices exhibit piezoelectric thin film thickness between one to three orders of magnitude smaller than the acoustic wavelength $\left(Z_{RUP}/\lambda \in \left[0.001;0.1\right]\right)$. This effective thickness to wavelength ratio is mostly favorable for the excitation and propagation of plate waves with mainly sagittal polarization, also known as Lamb waves. On the other side, the solidly mounted piezoelectric (i.e., not yet released) thin film constitutes an effective plate thickness equivalent to the substrate thickness (≈520 µm), and the thickness‐to‐wavelength ratio becomes unfavorable for both, Lamb wave and Surface Acoustic Wave (SAW) excitation, respectively.^[^ ^45^ ^]^

**Figure 4:**
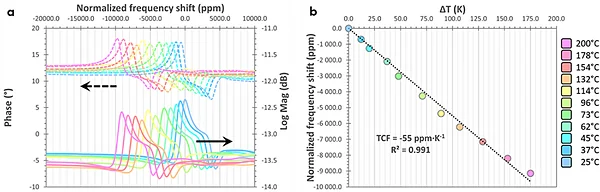
Study of the sensor perfomances of the devices: a) Frequency shift from both the magnitude and phase of the transmission between IDTs (S_21_) depending on the temperature applied to the device. b) Characterization of the Temperature Coefficient of Frequency (TCF) obtained by linear regression between the normalized frequency shift and temperature variation.

The transduction behavior from our self‐assembled 3D piezoelectric was further used to emphasize possible sensor applications. Consequently, sensor performances were quantified while measuring the transduction frequency shift according to the device temperature, from room temperature to 200 °C (**Figure** **4**). We observed a transduction frequency downward shift of the according peaks of both the phase and the amplitude characteristics, correlated with the temperature of the device. The thermal sensitivity comes from two cumulative effects, leading to a decrease of the corresponding transduction frequency: Firstly, the temperature increase leads to a decreased material's stiffness and thereby a reduced acoustic phase's velocity. Secondly, heating causes thermal expansion and thereby increases structure dimensions (i.e., λ). This thermal sensitivity is typically quantified by the means of a temperature coefficient of frequency (TCF), linking the transduction frequency (*F*_(*T*)_ [Hz]) to the variation of temperature (Δ*T* [K]), and a reference value taken at room temperature ($F_{\left(T_{0}\right)}$), with the following expression (Equation 3):

(3) $$F_{\left(T\right)}=F_{\left(T0\right)}\;\;\left(1+TCF\cdot \Delta T\right)$$

Then, the TCF can be graphically extracted by a linear regression between the frequency shift (Δ*F* [ppm]) and the variation of temperature (Equation 4):

(4) $$\frac{\Delta F}{F_{\left(T0\right)}}=\frac{F_{\left(T\right)}-F_{\left(T0\right)}}{F_{\left(T0\right)}}=TCF\cdot \Delta T$$

In our case, the thermal sensitivity from our self‐assembled nanomembrane piezoelectrics was characterized by a TCF value of −55  ± 0.5 ppm K^−1^ (Figure 4b).

**Figure 5:**
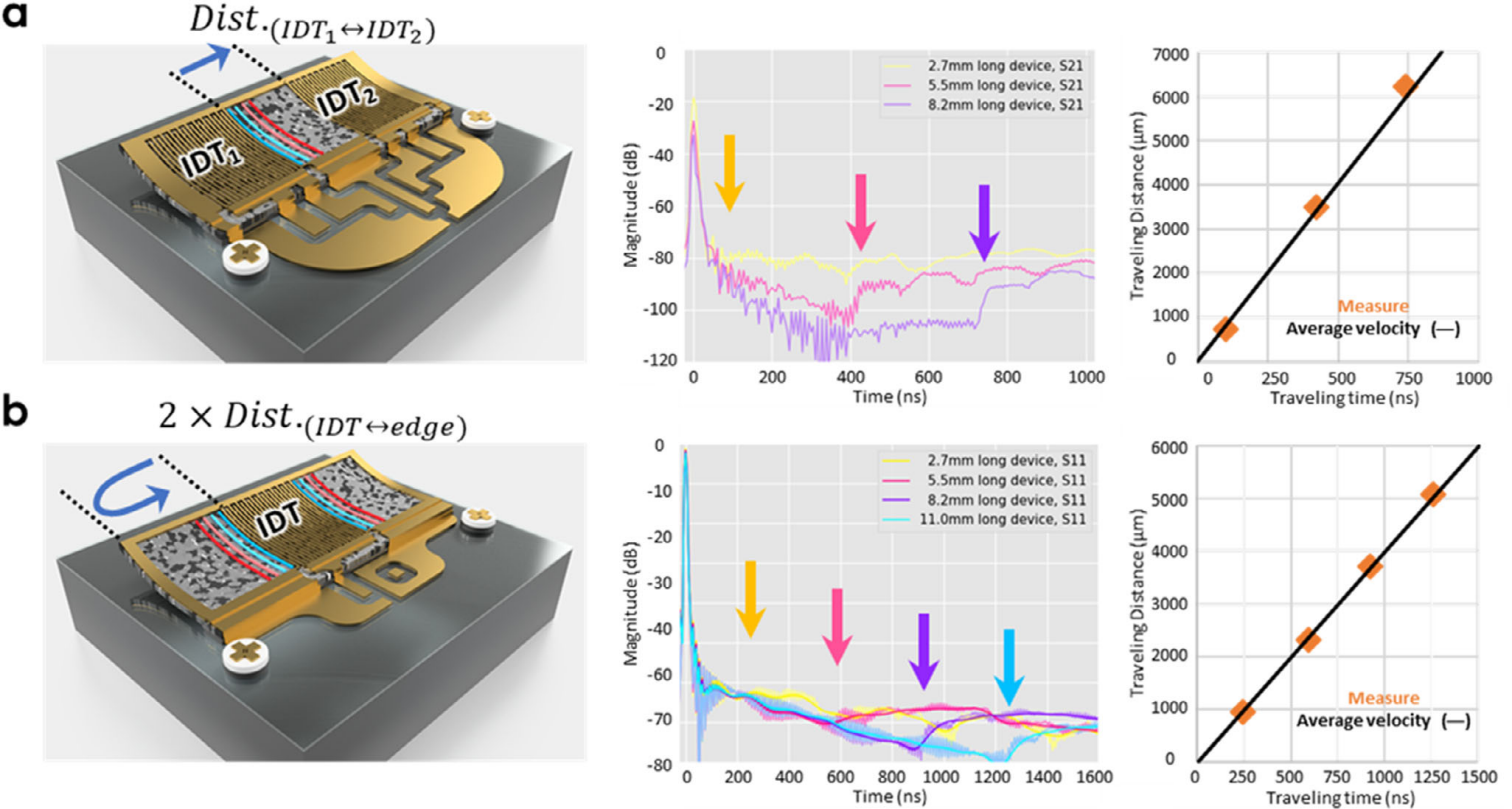
Electrical characterization of mechanical wave generation and propagation along the axes of the rolled‐up thin‐film piezoelectric devices, as measured in the time domain using either: a) 2‐port devices while measuring transmission delay from the S_21_ scattering parameters or b) 1‐port devices while measuring the “echo” delay from the S_11_ scattering parameters. (The tubular nanomembranes are represented here in a quasi‐unrolled state for better understanding of their structure with respect to acoustic wave propagation along the axial direction).

In a final series of experiments, we sought to characterize wave propagation within our devices as an initial approach to assess potential mechanical resonance phenomena of the fully 3D self‐assembled tubular nanomembrane. We analyzed the RF scattering (S‐) parameters via a vector network analyzer. However, this time, the data sets were converted from the frequency to the time domainvia the discrete Fourier transform, to analyze the time‐dependent electrical reflection and transmission of the devices, according to their functional architecture (**Figure** **5**). For that purpose, we employed two types of devices: first, a 2‐port delay‐line architecture (Figure 5a) with variable distance between the two IDTs (Figure S11, Supporting Information), and second, a 1‐port structure (Figure 5b) with a single IDT at the center of the structure, and variable length of the tubular nanomembrane (Figure S12, Supporting Information). With those two types of devices, having both variable tubular lengths, we respectively aimed at identifying either the delay of transmission or the delay of the first “echo” from the desired mechanical wave. The resulting time‐domain data were complex, yet they can be interpreted as follows. First, a strong signal appears quasi‐instantaneously at t_0_ and decays as fast as it arises. It is expected to be an undesired capacitive coupling which could be induced by the presence of the Ti seed layer acting as a floating electrode and propagating at the material inherent speed of light, similarly to other electric field‐related signals. The magnitude then decays while the mechanical wave travels along the tubular structure and then generates a jump of ≈+10 dB (highlighted by arrows) when reaching an IDT region. After that, the signal is too complex and difficult to interpret, especially considering all possible wave reflections at every electrode finger of the IDT. Despite the complexity of the time domain signal, we could correlate the mechanical transmission delay time to the geometry of the device, and especially the traveling distance (*d*_µ*m*_) before reaching again an IDT (Figure 5). To further validate our interpretations, we fabricated several control devices (2‐port architecture) with a “broken” mechanical transmission line, meaning that we generated two coaxial tubular structures out of each previous geometry (Figure S13, Supporting Information). Consequently, both transducers from the 2‐port architecture ended on a separate tubular nanomembrane while preserving the same electrode architecture as previously used for time domain analysis. The functionality of those control devices was confirmed with the presence of the peak on the electrical reflection coefficient (S_11_), and the experiment proved that energy transmission between IDTs (S_21_) requires a continuous tubular nanomembrane (Figure S14, Supporting Information).

With these results, we demonstrated that our self‐assembled tubular nanomembrane piezoelectrics are functional acoustic wave structures, being useful for actuators and sensors. Even though the vibration modes and mechanism of propagation have not yet been confirmed experimentally, we could foresee a Lamb/plate wave mode with excitation of the lowest‐symmetric mode (S_0_), considering the piezoelectric film thickness to wavelength ratio from our devices^[^ ^45^ ^]^ as well as the measured velocity compared to both the thickness to wavelength ratio and frequency thickness product.^[^ ^46^ ^]^ Preliminary measurements of surface‐normal vibration components as evidence for microacoustic mode excitation were attempted using a laser Doppler vibrometer (Figure S15, Supporting Information). An issue in these measurements is the relatively high field of depth of the vibrometry setup of several µm, leading to an integral measurement of the vibration over the whole wall thickness of the tubular structure. Despite of the limited measurement performance (comparatively low optical signal due to the reflection on the curved surface of the tubular structures made of thin films only) a lower frequency standing wave mode was detected. The monitored vibration remained localized within the boundaries of the IDTs and displayed a half‐wavelength compared to the transducer periodicity ( λ_*standing* *wave*_ =  λ_*IDT*_/2). This behavior might arise from a capacitive coupling with the Ti seed layer acting as a floating electrode, leading consequently to an equivalent circuit adapted from the modified Butterworth‐Van Dyke (MBVD) model for the electrically floating bottom surfaces^[^ ^47^ ^]^ with *L_m_*, *C_m_*,*R_m_* describing the acoustic response of the piezoelectric layer (Figure S16, Supporting Information). Meanwhile, the mechanical transduction identified above using a network analyzer (Figures 3, 4, 5) was not observed during vibrometry experiments. However, it was previously highlighted that the S_0_ Lamb wave mode results in a laterally vibrating mode when the film thickness is much smaller than the wavelength.^[^ ^46^ ^]^ Thus, the absence of measurable surface displacement for the identified transduction frequencies remains coherent if the vibration mode is the lowest Lamb wave symmetric mode (S_0_).

Despite the limited transduction performance from this first‐of‐its‐kind prototype, our self‐assembled tubular nanomembrane piezoelectrics could be greatly improved while tackling several loss effects as we previously highlighted.^[^ ^48^ ^]^ Furthermore, rolled‐up thin films have the particularity to display a planar geometry within the XZ projection plane and a curved one with quasi‐circular geometry in the YZ projection plane. Considering the particular geometry from the presented prototypes, our device should behave similarly to a classical planar Lamb wave resonator when the IDT's wavelength is oriented in the X_RUP_ direction (which is the case in the present study). Lamb wave resonators have already been particularly well studied and modelled^[^ ^46^, ^47^, ^49^ ^]^ including multilayer effects when the free‐standing thin film is a composite material.^[^ ^50^, ^51^ ^]^ In contrast, the boundary condition differs when considering wave propagation along the rolling length (Y_RUP_) and the possible winding‐to‐winding interaction once the rolled‐up structure is formed. Even though, analogous studies on Lamb wave propagation in pipes (i.e., at a larger scale) were previously completed in particularly for structural health monitoring and nondestructive testing applications.^[^ ^52^ ^]^ Finally, we can emphasize here an analogy with previous work performed on optical wave propagation in rolled‐up microstructures and whispering gallery resonators.^[^ ^15^, ^16^, ^17^ ^]^

Regarding technological constraints for device fabrication, there is mostly one key requirement. For the fabrication of functional rolled‐up devices, we require a precise and unidirectional release of the strained nanomembrane. A multidirectional release would lead to a large variety of free‐standing nanomembrane shapes and, inherently, to the nanomembrane rupture if the released surface exceeds a critical value, which depends on the curvature of the strain‐released nanomembrane.^[^ ^53^ ^]^ Notably, a higher nanomembrane release speed decreases the risks of mis‐assembly and allows for rolling a nanomembrane up to 1 cm long.^[^ ^19^ ^]^ The nanomembrane curvature is, in practice, stabilizing the free‐standing nanomembrane. An insufficient curvature would thus be a possible concern. In this study, we observed that shelf time from samples kept planar (prior to self‐assembly) could become an issue. In fact, after several months of shelf‐time, the planar multilayer thin film started to delaminate, and film buckling was observable, indicating that we could hardly make the SiN strain layer more compressive than it is here. This implies that the strain level could be the technological limit to fabricating rolled‐up devices with a thicker nanomembrane than presented here. Consequently, together with the fabrication constraints intrinsically linked to the choice of the sacrificial layer and release method,^[^ ^54^ ^]^ this led us to choose a dry release process based on a Silicon sacrificial layer, jointly with an ultra‐thin patterned passivation made of aluminium oxide to control the directional release.^[^ ^16^ ^]^ Besides, it has been previously highlighted that curvature direction would be affected by topological parameters^[^ ^55^, ^56^ ^]^ including the orientation of the interdigital electrodes.

## 3 Conclusion

With this work, we suggest a new approach to shape large piezoelectric nanomembranes into stable 3D self‐assembled tubular structures. The feasibility of this RUP concept was proven for devices with up to 10 mm^2^ of free‐standing active surface and a composite piezoelectric nanomembrane thickness ranging between 200 to 1500 nm. Compared to other previously published studies, our method allowed for the fabrication of functional microacoustic wave‐based devices, with a demonstrated working frequency between 50 MHz up to ≈1 GHz and respective transducer wavelengths (i.e., periods) between 160 µm down to 8 µm. Despite their relatively low operational performance, we confirmed sensor application feasibility similar to other piezoelectric devices. The piezoelectric excitation, propagation, and transmission of an acoustic wave mode within a free‐standing tubular nanomembrane have been demonstrated. By improving the performance of the piezoelectric layer and further investigation of the peculiar acoustic excitation and propagation, our 3D self‐assembled tubular piezoelectrics would find applications in telecommunication, energy harvesting, and acoustofluidics. Furthermore, the 3D geometry and self‐assembly process would enable new topological designs for fundamental research on acousto‐optic and magneto‐acoustic phenomena.

## 4 Experimental Section

### Sample Fabrication—Laboratory Environment

All fabrication processes, with the only exception for sputtering processes (Ti‐AlN layer and Cr‐Au metallization), were done in a clean‐room environment (ISO 7).

### Sample Fabrication—Substrate cleaning

Substrates: Silicon‐On‐Insulator wafer (SOI), handling layer: 620 µm, buried thermal oxide (BOX): 1 µm, device layer of 2.5 µm < 100 > N/Ph, R < 5 Ω, (ULTRASIL LLC, Hayward, CA, USA).
Sample cleaning: Samples were first cleaned by successive immersion and sonication in two baths of acetone, and one of isopropyl alcohol (VWR, Darmstadt, Germany), then immediately dried under compressed nitrogen. Finally, a surface cleaning was performed under O_2_‐plasma for 30 min (Pico, Diener electronic GmbH, Ebhausen, Germany).

### Sample Fabrication—Lithography

Resists: either AZ‐5214e for lift‐off processes or AZ‐4562 as a dry‐etching mask
Exposure: Alignment and exposure were done with a mask‐less aligner (MLA‐100, Heidelberg Instruments, Germany).
Development: All photoresist development processes were done using sodium‐based developers (TMAH free) to minimize attack on Al‐containing layers, using either undiluted AR‐300‐35 (AllResist, Germany) or 1:1 diluted AZ Developer MIC (Microchemicals GmbH, Germany).

### Sample Fabrication—Atomic Layer Deposition

Key step in the thin film rolling feasibility: three different processes were required to control the pinhole‐free encapsulation of the thin film stack while enabling proper electrical connections (see Figure S1, steps 1, 7, and 9, Supporting Information). Deposition consisted in a thermal ALD process using TMA precursor and H20 (GEMStar XT‐P, Arradiance, USA). Each cycle of atomic layer deposition involved 5x consecutive pulses of precursor (21 ms pulse, 3 s delay) a purge (15 s), and then 5x consecutive pulses of water (21 ms pulse, 3 s delay) and a purge (20 s) to conclude it. The first process (Figure S1 step 1, Supporting Information) consisted in a 40x cycles process at 280 °C, resulting in ≈4 nm of AlOx. The second process (Figure S1 step 7, Supporting Information) consisted in a 100x cycles process at 120 °C using an AZ‐4562 mask for sonication‐assisted liftoff‐like patterning and passivation of the vertical edges from the thin film stack. The third and last process (Figure S1 step 9, Supporting Information) consisted in a 20x cycles process at 280 °C, resulting in a continuous and pinhole‐free film of ≈2 nm AlOx.

### Sample Fabrication—Plasma‐Enhanced Chemical Vapor Deposition

The highly compressive SiN layer required to induce rolling of the thin film stack was deposited using PE‐CVD (ICPECVD SI 500 D, Sentech, Germany). The deposition was run at 280 °C with 1200 W ICP power, −75 V bias voltage, with a reactor pressure set at 3 Pa and using 250 sccm SiH gas precursor together with 80 sccm N_2_ and 140 sccm Ar.

### Sample Fabrication—Sputtering

Piezoelectrics: Aluminum nitride and its seed Titanium layer were deposited using the same deposition tool (HZM‐4P, Von Ardenne, Germany) and a multi‐step deposition within a single process. First, a 60 nm Titanium seed layer was deposited by DC sputtering at 1000 W power, at room temperature, 5 mTorr chamber pressure, and under 27 sccm Ar gas flow for 2 min 30 s (24 nm min^−1^). Then, the Wurtzite fiber texture Aluminum Nitride was deposited by DC‐reactive sputtering at 500 W power, at room temperature, 5 mTorr chamber pressure, and under 27 sccm Ar and 27 sccm N_2_ gas flow at a deposition rate of ≈3,1 nm min^−1^. (NB: 6″ Al and Ti targets with 5N purity and a target to substrate distance of ≈6 cm).
Metallization: Electrodes for the transducer (IDT (Figure S1 step 4, Supporting Information) and contact pads (Figure S1 step 8, Supporting Information)) were patterned by lift‐off with chromium and gold deposited by DC sputtering (nanoPVD, Moorfield nanotechnology, UK). CrAu layer thicknesses were respectively 10 nm Cr with 50 nm Au for the IDTs and 10 nm Cr with 100 nm Au for the contact lines and pads.

### Sample Fabrication—Dry Etching Processes

Nanomembrane patterning (Figure S1 step 5, Supporting Information): the multi‐layer stack of AlN‐Ti‐SiN‐AlOx (top‐down) was dry‐etched using chlorine chemistry on a RIE (PlasmaLab 100 RIE, OXFORD Instruments, UK) at ≈0,5 nm s^−1^ while using ≈8 µm thick AZ‐4562 mask. For better temperature control, samples were glued to the carrier using PMMA and later detached using Acetone. (Detailed parameters = P: 0015 mBar, Ar: 20 sccm, BCl_3_: 20 sccm, Cl_2_: 10 sccm, ICP Power: 750 W, RF Power: 150 W, DC Bias: ≈350 V, T: 30 °C).
Sacrificial layer patterning (Figure S1 step 6, Supporting Information): the Si sacrificial layer (device layer from the SOI substrate) was dry‐etched using fluorine chemistry and cryogenic process on a reactive ion etching tool (PlasmaLab 100 RIE, OXFORD Instruments, UK) at > 35 nm s^−1^ while using a crosslinked AZ‐5214e mask. For better temperature control, samples were glued to the carrier using PMMA and later detached using Acetone. (Detailed parameters = P: 0010 mBar, SF_6_: 70 sccm, O_2_: 6 sccm, ICP Power: 450 W, RF Power: 40 W, DC Bias: ≈30 V, T: ‐120 °C).
Release‐window opening (Figure S1 step 10, Supporting Information): the window in the AlOx passivation layer was opened using chlorine chemistry on a RIE (PlasmaLab 100 RIE, OXFORD Instruments, UK) at ≈0,5 nm s^−1^ while using ≈8 µm thick AZ‐4562 mask. 45 s of etching was done to grant the complete removal of the AlOx on the window. For better temperature control, samples were glued to the carrier using PMMA and later detached using Acetone. (Detailed parameters = P: 0015 mBar, Ar: 20 sccm, BCl_3_: 20 sccm, Cl_2_: 10 sccm, ICP Power: 750 W, RF Power: 150 W, DC Bias: ≈350 V, T: 30 °C)
Nanomembrane release and rolling (Figure S1 step 11, Supporting Information): the multi‐layer membrane was released using fluorine chemistry on a RIE (PlasmaLab 100 RIE, OXFORD Instruments, UK). For better temperature control, samples were glued to the carrier using PMMA and later detached using Acetone. The release progress was monitored with a standard microscope by removing the sample every ≈30 min from the chamber. (Detailed parameters = P: 0076 mBar, SF_6_: 70 sccm, O_2_: 2 sccm, ICP Power: 750 W, RF Power: 0 W, DC Bias: 0 V, T: 0 °C).
NB: Some of the first samples were released using XeF_2_ dry etching (Xactix e2, SPTS, UK) to allow for live monitoring during thin film rolling (see Movie S1, Supporting Information).

### Sample Characterization—X‐Ray Diffraction (XRD)

Thin films and more specifically TiAlN nanomembrane were characterized on a Panalytical X‘PertPro MRD diffractometer (Cu‐Kα). Pole figures acquisition parameters are detailed in Figure S4 (Supporting Information).

### Sample Characterization—Transmitted Electron Diffraction (TEM)

To prepare TEM *lamellae*, the samples were embedded in polymer M‐Bond 610 by vacuum infiltration and mildly baked for hardening. From the embedded sample, thin slices were saw cut to expose the tube. No further preparation specific for polymers (heavy metal staining & hardening) was applied. A thin Cr deposit was applied to reduce charging. *Lamellae* were prepared by FIB cut in a FIB/SEM NVision40 (Carl Zeiss, Germany) using Ga ions with E = 30 and 5 kV for final polishing. TEM, STEM, and EDX were performed on a Tecnai F30 (FEI) operating at 300 kV.

### Sample Characterization—Imaging

Microscopy: Images of samples were recorded with a Keyence VHX‐5000 digital microscope

### Sample Characterization—Strain Measurement

The strain was obtained by measuring the bow of a Si wafer (diameter 4′’, thickness 525 µm) before and after the deposition of the thin film on a Dektak XT (Bruker).

### Sample Characterization—Electrical Characterization and Data Treatment

Electrical characterizations were performed on a Cascade Microtech 150 mm Probe Station while using a pair of Z‐Probe (Cascade Microtech # Z 040 K3N GSGSG 200) and coaxial cables for signals DC to 65 GHz (Cascade Microtech #161‐099). The measurements were acquired on a performance network analyzer (Keysight PNA‐N5227A) after appropriate 2‐port calibration with a reference sample from Suss‐Rosenberger (# CSR‐32 GSGSG 200). The IDT wavelength‐dependent transduction frequency (Figure 3) was analyzed and post‐treated using the SciKit‐RF toolkit in Python. The scattering parameters were gated (center: 0; span: 100 ns) to separate and subtract the electromagnetic coupling contribution from the original information and consequently isolate piezoelectric coupling information. The thermal sensitivity measurement (Figure 4) was performed while using a Peltier element to control temperature above room temperature (humidity and condensation were an issue below room temperature). The temperature of the device was acquired with a contactless infrared thermometer directly on the device substrate. The temperature measurement quality was further controlled while observing a linear correlation between the measured temperature and the power applied to the Peltier element (with a regression coefficient > 0.99). Here, the maximum value of the log from the magnitude was used for frequency shift tracking without priori data treatment. Finally, Time domain signals (Figure 5) were obtained with a measurement window centered on the transduction frequency and 150 MHZ span. They were analyzed using SciKit‐RF again to convert frequency domain measurements to time domain.

### Sample Characterization—Statistical Analysis

In this work, presented statistical analysis always consists in mean values, the presented errors are the standard deviations, and the sample size is supplied.

## Conflict of Interest

The authors declare no conflict of interest.

## Supporting information



Supporting Information

Supplemental Movie 1

Supporting Information

## Data Availability

The data that support the findings of this study are available from the corresponding author upon reasonable request.
