# Supplementary material for: Self‐Assembled Inorganic Nanomembrane Tubes: Rolled‐Up Piezoelectrics for Microacoustic Wave‐Based Actuators and Sensors
Source: Adv Mater. 2025 Nov 28;38(51):e12619. doi: 10.1002/adma.202512619 (PMC13568921; doi:10.1002/adma.202512619)
Supplement: Supplementary file 1 [file ADMA-38-e12619-s002.docx]

Supporting Information

Self-assembled inorganic nanomembrane tubes: rolled-up piezoelectrics for microacoustic wave-based actuators and sensors

Raphaël C. L-M. Doineau*, Christian N. Saggau, Stefan Baunack, Thomas Gemming, Yara Abdelaal, Robert Weser, Oliver G. Schmidt, Hagen Schmidt, Andreas Winkler, Mariana Medina-Sánchez*

Device fabrication process overview:

The fabrication and self-assembly of tubular nanomembrane piezoelectrics can be summarized in the following 11-step process flow (Figure S1). The CAD drawing (Figure S17) used for UV- and electron beam lithography can be found in the supporting files. Process points for deposition and etching are detailed in the Experimental section of the paper.

First, we built the material stack on top of a SOI wafer (**Figure S1a**) and successively deposit thin-films of AlO_x_, SiN, Ti and AlN (**step 1 to 3**), with AlO_x_ acting as etch passivation layer (a protection barrier against the SF_6_ or XeF_2_ etchant used at the end for the release and self-assembly), with SiN acting as strain layer (highly compressive strain), Ti acting as a seed layer for AlN the functional piezoelectric layer (i.e.: Ti-AlN layers have a slightly tensile strain).

Then a top-down patterning is applied (**Figure S1b**) first to structure the interdigital transducer (**Step 4**), second to shape the nanomembrane by a single etch process, consecutively etching AlN, Ti, SiN, and AlOx thin films until the sacrificial layer is reached (**Step 5**), and finally etching sacrificial layer to keep it only below the nanomembrane area (**Step 6**).

The devices are then further functionalized (**Figure S1c**) by first a side passivation with AlO_x_ thin film (**Step 7**) used both to electrically isolate the vertical thin edge from the nanomembrane, avoiding an electrical shortcut between later contact lines and the Ti-layer, and to reinforce passivation performance against pinhole etching issues (see later details on defects possibility). The electric contact pads are structured on the sacrificial layer free area with contact lines crossing over the passivated edge of the nanomembrane to reach the interdigitated transducer (Step 8). Finally, a thin AlO_x_ passivation layer is deposited on all surfaces of the device (**Step 9**). Lastly, two consecutive steps are required for the nanomembrane self-assembly (**Figure S1d**): an etching window is opened on the opposite side to the electrical contact pads (**Step 10**) to ensure a clean and linear etch front when releasing the nanomembrane with a dry etching process (**Step 11**).

The nanomembrane release (**Step 11**) with XeF_2_ etching was monitored with a camera acquisition at the end of every etching cycle (See **Suppl. Mov. 1**). This helped us to identify a passivation layer weakness along the nanomembrane edge that should remain anchored to the substrate (i.e. edge with electrode contacts). This issue was finally solved with an increase of the related passivation layer thickness (**Step 7**) up to 100x cycles of ALD for an efficient (i.e. pinhole-free) passivation but still thin enough for a feasible sonication-assisted lift-off process.

With this process flow, nanomembranes curl and roll into steady tubular structures with a curvature inversely correlated to the thickness ratio between the slightly tensile thin film (Ti-AlN) and the highly compressive SiN layer (**Figure S2**). Digital microscopy images of the devices used for the data acquisition of the present study are shown here (**Figure S3**).

**Figure S1.** *Graphical illustration of the sample fabrication process with an 1- step workflow consisting of either a material deposition or material etching. A majority of those process steps required UV-lithography for functional patterning (i.e.: Step 4 to Step 8 and Step 10) with a total of 6 masks to achieve a complete device fabrication. All PVD processes consist here in DC-Sputtering.*

**Figure S2.** *Demonstration of the controllability of the rolled-up curvature: a) representation of the rolled-up tubular diameter as a function of SiN strained layer thickness and Ti-AlN layer thickness and a surface fitting. Sample pictures acquired on a digital microscope showing rolled-up tubular structure b) with a consistent diameter for devices from the same batch c) with a lowest achieved curvature (r_min_) of 32 µm and d) and largest achieved curvature(r_max_) of ~750 µm.*

**Figure S3.** *Sample pictures acquired on a digital microscope highlighting a) the longest achieved tubular structure (Y_RUP,max_) being 11 mm and b) and the longest achieved rolling distance (X_RUP,max_) being 3.5 mm. The samples imaged here are a) the 1-port resonator device and c) 2-port delay line used for the monitoring of mechanical wave propagation and d) the sample used for sensor performance analysis*

Material characterization supplementary data:

In the following figures (**Figure S4-S6**), we present the detailed crystalline texture from the AlN thin film anchored to the substrate (**Figure S4**) as well as after tubular self-assembly (**Figure S6**). The material composition from the rolled-up nanomembrane was measured by STEM-HAADF (**Figure S5**).

**Figure S4.** *Detailed Pole figure acquisition parameters*

**Figure S5.** *Composition analysis by STEM-EDS. (a) STEM-HAADF image of a part of the layer stack, (b) Variation of elemental intensities along the line in (a). The STEM-HAADF intensity is shown for orientation.*

**Figure S6.** Fig. *Analysis of crystallinity by SAED in different regions of the AlN layer. Upper row TEM bright filed images with the area for SAED marked. Lower row SAED patterns in the marked regions prove the single crystalline structure of the investigated areas.*

Device electrical characterization and experimental design supplementary information:

In the following figures (**Figure S7-S17**), we present supplementary electrical characterizations as well as piezoelectric transduction measurement and device architecture developed for the present study. As a starting point (**Figure S7**), we emphasize the inversely proportional linear relation between the IDT’s wavelength and the transduction frequency showing a quasi-uniform propagation velocity.

**Figures S8, S9 and S10** show batch performance of Rolled-Up Piezoelectrics (**Figure S8**) along with the results obtained with two types of negative control designs: first with “planar” devices (i.e.: prior free-standing nanomembrane self-assembly) and second with devices made with an amorphous AlN layer (i.e.: without piezoelectric AlN crystalline texture). Both negative control experiments demonstrate the absence of measurable piezoelectric transduction.

**Figures S11 & S12** illustrate the design architecture from our 1-port transducer (**Figure S12**) and 2-port delay line (**Figure S11**) along with the wave propagation path (black arrows) according to the length of the rolled-up structures.

**Figures S13 & S14** demonstrate that the transmission of the signal in a 2-port delay line architecture is inherent to a mechanical wave propagation mechanism. For this demonstration, a device with a discontinuous transmission line has been engineered and illustrated in **Figures S13**. The electrical characterization of the control devices (**Figures S14**), shows a piezoelectric transduction at the first IDT (see |S_11_| reflection coefficient signal) but an absence of transmission to the second IDT of the “broken” delay line (see |S_21_| transmission coefficient signal).

**Figures S15** presents the results obtained with the laser doppler vibrometer. With this equipment we only monitored a surface displacement response with a measured wavelength corresponding to half of the IDT’s wavelength. Furthermore, the measured displacement shows only a standing wave pattern.

**Figures S16** presents an electrical equivalent circuit that could support this half wavelength behavior.

**Figures S17** illustrates the CAD designs and lithography masks that have been used for the present study (see also **Supplementary CAD file**).

**Figure S7.** *Correlation between IDT’s wavelength and time period at transduction frequency and practically uniform acoustic velocities.*

**Figure S8.** *Scattering parameters in terms of single IDT reflection (S_11_) and transmission between IDTs (S_21_) from height distinct devices from a single batch with nanomembrane released from the substrate (i.e. as a “RUP”)*

**Figure S9.** *Scattering parameters in terms of single IDT reflection (S_11_) and transmission between IDTs (S_21_) from height distinct devices from a single batch prior to release from the substrate (i.e. as a planar anchored thin film)*

**Figure S10.** *Scattering parameters in terms of single IDT reflection (S_11_) and transmission between IDTs (S_21_) from height distinct devices from a single batch made with amorphous and consequently non-piezoelectric AlN*

**Figure S11.** *Schematic illustration of the three different designs of 2-port delay lines with respective transmission distances being 820 µm, 3 570 µm and 6 320 µm.*

**Figure S12.** *Schematic illustration of the four different designs of 1-port “echo” devices with transmission distances (“2.d_µm_”) being respectively* $(2\times990)$ *µm,* $(2\times2 365)$*µm,* $(2\times3 740)$ *µm and* $(2\times5 115)$ *µm.*

**Figure S13.** *Schematic illustration of the mechanical discontinuity within the transmission line (left) and digital microscopy images of the produced device (right).*

**Figure S14.** (a,b) *Scattering parameters in terms of single IDT reflection (S_11_) and (c,d) transmission between IDTs (S_21_) as well as (e,f) time domain data from devices with a continuous transmission line (left) or a discontinuous (broken) transmission line (right).*

**Figure S15.** *Vibrometer measurement performed on the curved top surface of the rolled-up nanomembranes on two devices with a respective IDT wavelength of 40 µm (left) and 80 µm (right). Surface displacement amplitudes have been measured punctually with a frequency scan (a,b) and spatially along the surface of the device at fixed frequency (c,d) of respectively 20.375 MHz and 9.016 MHz.*

**Figure S16.** *(a) Illustration of the nanomembrane cross-section of AlN rolled-up structures with floating bottom surface and (b) simple physical model for the capacitive contributions in the one-port structure and the equivalent C_0_ in the modified Butterworth-Van Dyke model*

**Figure S17.** CAD design export view used for planar device fabrication: *(a) a single nanomembrane microstructure within a 3 mm by 3.25 mm device footprint before self-assembly, (b) electrode design details from a 2-port delay line with the thin metallization (light colors) on top of the piezoelectric nanomembrane (grey) and with contact lines (dark colors) anchored on the substrate for 2-port probe station measurement (yellow), and (c) 2-port delay lines with continuous (left) or discontinuous (right) nanomembrane or (d) 1-port structure with centered IDT (left) or off-centered IDT (right), both with variable propagation distances used for the monitoring of mechanical wave propagation.*
